# Assessment of the FreeStyle Libre 2 interstitial glucose monitor in hypo‐ and euglycemic cats

**DOI:** 10.1111/jvim.16820

**Published:** 2023-08-03

**Authors:** Alisa S. Berg, Chiquitha D. Crews, Christopher Adin, Adriana Alfonso‐Castro, Susan B. Hill, Jocelyn Mott, Chen Gilor

**Affiliations:** ^1^ Department of Small Animal Clinical Sciences, College of Veterinary Medicine University of Florida Gainesville Florida USA

**Keywords:** cat, continuous glucose monitor, diabetes, euglycemia, feline, flash glucose monitor, FreeStyle Libre 2, hyperglycemia, hypoglycemia, insulin, interstitial glucose monitor, veterinary

## Abstract

**Background:**

Continuous glucose monitoring systems have been validated for eu‐ and hyperglycemic cats. The FreeStyle Libre 2 (FSL2) is sufficiently accurate in people during hypoglycemia to guide critical treatment decisions without confirmation of blood glucose concentration (BG).

**Objectives:**

Assess FSL2 accuracy in cats with hypoglycemia.

**Animals:**

Nine healthy, purpose‐bred cats.

**Methods:**

Hyperinsulinemic‐hypoglycemic clamps were performed by IV infusion of regular insulin (constant rate) and glucose (variable rate). Interstitial glucose concentration (IG), measured by FSL2, was compared to BG measured by AlphaTrak2. Data were analyzed for all paired measurements (n = 364) and separately during stable BG (≤1 mg/dL/min change over 10 minutes). Pearson's r test, Bland‐Altman test, and Parkes Error Grid analysis respectively were used to determine correlation, bias, and clinical accuracy (*P* < .05 considered significant).

**Results:**

Overall, BG and IG correlated strongly (r = 0.83, *P* < .0001) in stable glycemia and moderately at all rates of change (r = 0.69, *P* < .0001). Interstitial glucose concentration underestimated BG in euglycemia, but the BG‐IG difference was progressively smaller as BG decreased (12.9 ± 12.2, 8.8 ± 11.2, −3.2 ± 7.4, and −7.8 ± 5.2 mg/dL in the ranges of 80‐120 [n = 64], 60‐79 [n = 29], 50‐59 [n = 71], and 29‐49 mg/dL [n = 53], respectively).

**Conclusions:**

Although IG underestimates BG throughout most of the hypo‐euglycemic range, IG generally overestimates BG in marked hypoglycemia (<60 mg/dL). It is therefore imperative to evaluate FSL2 results in this critical range with caution.

**Abbreviations**
BG: blood glucose
CGM: continuous glucose monitor
FSL: FreeStyle Libre
FSL2: FreeStyle Libre 2
IG: interstitial glucose
VAP: vascular access port

## 1 INTRODUCTION

Continuous, minute‐by‐minute interstitial glucose monitors (CGMs) have revolutionized the monitoring of insulin treatment. They allow for evaluation of glucose trends over time while decreasing overall cost of care and blood sampling‐associated patient discomfort.^1^ Their use improves detection of hypoglycemia in human^2^ and veterinary patients, providing a means to ameliorate primary stressors reported by owners.^3^, ^4^ The first generation of the FreeStyle Libre monitor (FSL1; Abbott Laboratories) has been validated for use in veterinary patients in both the inpatient and outpatient setting.^5^, ^6^, ^7^, ^8^, ^9^, ^10^, ^11^

The accuracy of the FSL1 has been evaluated previously in both healthy and diabetic cats, where it correlates well with blood glucose concentration (BG) in the eu‐hyperglycemic range (r = 0.90‐0.97),^1^, ^5^, ^7^, ^12^ but underestimates BG (in healthy cats) by a fixed mean of 23 mg/dL in these glycemic ranges.^7^, ^12^ The correlation of BG and interstitial glucose concentration (IG) as measured by the FSL1 is reported to be much lower in hypoglycemia, but interpretation is limited by very small sample sizes (n = 4,^7^, ^12^ n = 6,^1^ n = 9,^12^ and n = 10^5^ paired IG samples at BG < 70 mg/dL). This discrepancy is of particular concern in diabetic cats, in which a subset might be unable to effectively compensate for hypoglycemia because of impaired counter‐regulatory responses, as seen in canine, human, and rodent diabetic patients.^13^

The FreeStyle Libre 2 (FSL2), like its predecessor, has a compact design, simple sensor application, smartphone integration, and factory calibration technology that obviates the need for daily BG sampling.^1^, ^5^ In people, the FSL2 is more accurate in the hypoglycemic range and can be used without BG confirmation to make treatment decisions, unlike the FSL1.^14^ Although the FSL2 has been used in cats with anecdotal reports of clinical success, it should be assessed at all levels of glycemia. Extrapolation from data in humans risks erroneous clinical decision‐making, which is of greatest consequence in the potentially life‐threatening hypoglycemic range.

Our primary objective was to determine FSL2 accuracy in cats with hypoglycemia. We hypothesized that IG would underestimate BG during periods of stable hypoglycemia with similar or decreased accuracy to what has been reported in euglycemia and hyperglycemia.

## 2 MATERIALS AND METHODS

### 2.1 Animals

All animal use was approved by the University of Florida Institutional Animal Care and Use Committee (protocol number 202011101). Nine neutered, purpose‐bred, domestic shorthair cats (5 male, 4 female) were included, with median (range) ages of 6 (5, 6) years, respectively. Median (range) body condition score was 6 (5‐7) on a 9‐point scale. Median (range) body weight was 4.9 kg (4.0‐7.1 kg). Cats were group‐housed in facilities accredited by the Association for Assessment and Accreditation of Laboratory Animal Care (AAALAC) International. All cats were socialized and acclimatized to catheter bandages and routine handling and restraint for at least 3 months before the start of the study. Extensive environmental enrichment was provided, including 1 to 3 hours of daily interaction with humans and 24‐hour access to various toys and climbing apparatus. Cats were fed commercial dry cat food (Envigo 2060 Teklad Global Cat Diet) ad libitum in sufficient amount to maintain body weight. Cats were deemed healthy based on routine weekly physical examinations, annual systemic blood tests (CBC and serum biochemistry panels), and the absence of clinical signs of disease. Experiments were performed at ambient temperatures between 20 and 24 C in the cats' routine environment.

### 2.2 Vascular access port and peripheral catheter placement and maintenance

A vascular access port (VAP, CompanionPort, CP 202 K, Norfolk Vet Products, Skokie, Illinois) was surgically placed under general anesthesia into the jugular vein of each cat at least 3 months before beginning the experiment. Vascular access port patency was maintained by weekly heparinized saline (10 U/mL) irrigation followed by a 0.5 mL (100 U/mL) heparin lock injection, which was aspirated and discarded before sample collection. The night before each experiment, a peripheral IV catheter (3/4″ 22‐24ga, Terumo Sur‐vet Surflo ETFE, Ontario, Canada) was placed in a cephalic vein and removed at the end of the procedure. This cephalic catheter was used exclusively for IV infusions. For placement of cephalic catheters, cats were sedated using dexmedetomidine (5‐15 μg/kg, IM). Sedation was reversed using a dexmedetomidine‐equivalent volume of IM atipamezole (50‐150 μg/kg) and the cats monitored until fully recovered from sedation.

### 2.3 Paired blood and interstitial glucose measurements

Blood glucose concentrations were measured by a hand‐held glucometer validated for use in cats (AlphaTrak2, Blood Glucose Monitoring System, Zoetis, Parsippany, New Jersey).^15^ A FSL2 sensor (Abbott Laboratories, Abbott Park, Illinois) was placed at least 1 hour before each procedure. Interstitial glucose concentration was measured by scanning the FSL2 at the same time as each BG measurement and both were recorded as paired values. Glucose and insulin were infused exclusively via a peripheral cephalic IV catheter while all blood sampling was performed only via the jugular VAP.

### 2.4 Hyperinsulinemic‐hypoglycemic clamps

Controlled hypoglycemia was achieved using a hyperinsulinemic‐hypoglycemic clamp protocol, modified from what has been described in dogs.^13^ Blood glucose and IG concentrations were measured at baseline (time −10 and 0 minutes) before initiating the clamp procedure. Hypoglycemia was induced by IV infusion of regular insulin (Humulin R, Lilly, USA) at a constant rate (0.1 U/kg/h) using a syringe pump (Medfusion 3600, Medfusion, Inc, North Carolina, USA). Glucose (Dextrose 50% [Vet One, Vedco, or Durvet], diluted to 20% with sterile 0.9% saline) was infused at a changing rate, adjusted every 5 minutes based on BG measurements, to achieve and maintain glycemic clamp targets. Regular insulin infusion was commenced at time 0 to allow BG to decrease without concurrent glucose infusion. After BG decreased at least 10% from baseline, glucose infusion was commenced and its rate adjusted to decrease BG to a target of 60 mg/dL over 30 minutes. Glucose rate then was adjusted to maintain BG within 10% of the first glycemic target (54‐66 mg/dL) for 60 minutes. After 60 minutes of appropriate glycemic clamping at the first target, the glucose infusion rate was decreased to allow BG to decrease to a target of 45 mg/dL over 30 minutes. Glucose rate then was adjusted to maintain BG within 10% of this target (41‐49 mg/dL) for an additional 60 minutes. After maintenance of the 45 mg/dL glycemic clamp, insulin infusion was discontinued and glucose infusion rate gradually decreased until euglycemia returned without glucose supplementation.

Additional blood sampling was performed at baseline and at each clamped BG as part of a separate study in which counter‐regulatory hormones were measured. Less than 20 mL of blood was drawn from each cat to account for these 10 samples (1.6 mL each) and all BG samples (0.05 mL each). This total volume accounts for ≤4% of total blood volume and therefore was deemed unlikely to affect the results of the study.

### 2.5 Data and statistical analysis

Data first were analyzed from all time points in which concurrent measurement of BG and IG were available. Analysis was repeated on the data subset limited to stable BG to account for blood‐interstitium lag time.^7^, ^12^ Stable glycemia was defined as a change in BG of ≤1 mg/dL/min over 10 minutes preceding sample acquisition. Average absolute change first was calculated between 2 consecutive BG measurements 5 minutes apart (using the formula: [BG(*t*_*i*_) − BG(*t*_*i*−5_)]/5 in which *t*_*i*_ is time point *I* and *t*_*i*−5_ is the time point preceding it). Average change over 10 minutes then was calculated by averaging the rate of change in the previous 2 5‐minute periods. If a 5 minute interim data point was missing, the rate of change was averaged directly between 2 readings 10 minutes apart ([BG(*t*_*i*_) − BG(*t*_*i*−10_)]/10). Such was the case in a single IG reading per cat, for a total of 9 data points throughout the entire data set.

Data were assessed for normal distribution using the Shapiro‐Wilk test. The correlation between BG and IG was assessed using Pearson's r test and difference between glycemic groups compared using an analysis of variance (ANOVA) with Dunnett correction for multiple comparisons (with the 80‐120 mg/dL glycemic range as control). Data homogeneity of variance was verified using Brown‐Forsythe and Bartlett's tests. Proportional bias between BG and IG measurements was assessed using the Bland‐Altman test. Accuracy was assessed according to ISO 15197:2013 guidelines, with acceptable analytical accuracy defined as 95% of IG results being within 15 mg/dL (when BG ≤100 mg/dL) or 15% (when BG >100 mg/dL) of paired BG, and clinical accuracy as ≥99% of IG falling in zones A or B of the Parkes Error Grid analysis as formulated most strictly for people with Type I diabetes.^16^ Analytical accuracy also was assessed by calculating relative differences for each time point (100 × [IG − BG]/BG) as previously described^17^ and then calculating mean absolute relative difference (MARD), median absolute relative difference (mARD), mean relative difference (MRD), and mean absolute difference (MAD) for the entire data set and for data at stable BG. Statistical significance was defined as *P* < .05.

## 3 RESULTS

**FIGURE 1:**
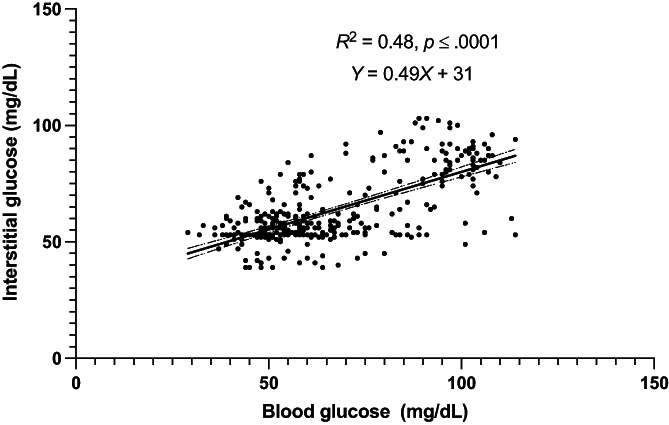
Correlation between blood glucose (BG) and interstitial glucose (IG) concentrations in healthy cats (n = 9) in hypo‐ and euglycemia at all rates of glycemic change. The solid line represents best fit with dashed lines representing the 95% CI of the best fit. Blood glucose measured by AlphaTrak2. Interstitial glucose measured by FreeStyle Libre 2 continuous interstitial glucose monitor.

**FIGURE 2:**
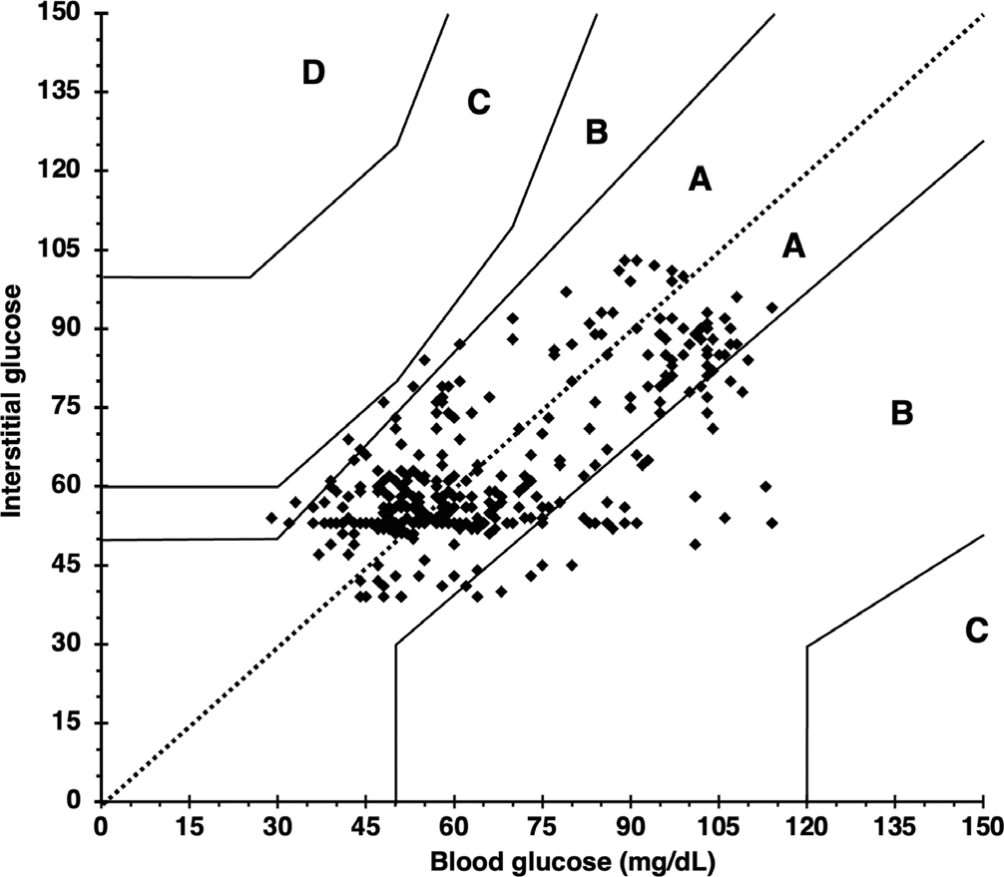
Parkes Error Grid analysis exhibiting excellent clinical accuracy of FSL2 at hypo‐ and euglycemia in healthy cats (n = 9) at all rates of glycemic change. All data points fall within zone A (indicating no change in clinical action) or zone B (indicating change in clinical action unlikely to affect outcome), with 91.5% (n = 333) in A and 8.5% (n = 31) in B. BG, blood glucose as measured by AlphaTrak2. IG, interstitial glucose as measured by FSL2, FreeStyle Libre 2 continuous interstitial glucose monitor.

**FIGURE 3:**
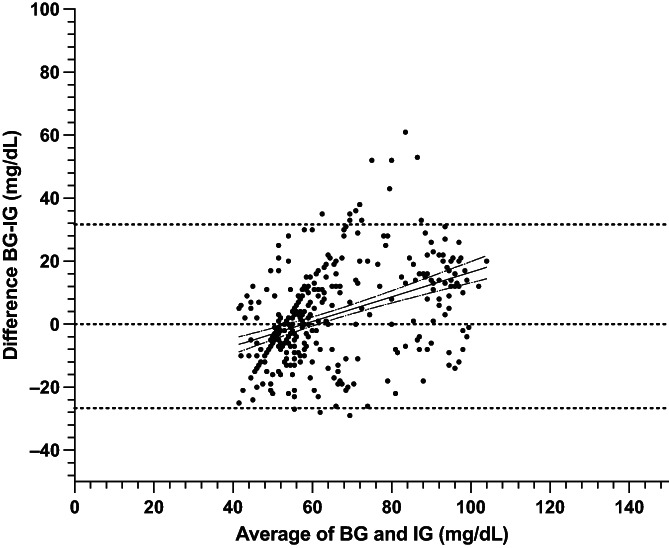
Bland Altman plot of agreement between blood glucose (BG) and interstitial glucose (IG) concentrations in hypo‐ and euglycemia in healthy cats (n = 9) at all rates of glycemic change. The middle, solid line represents best fit with dashed lines representing the 95% CI of the best fit. Blood glucose measured by AlphaTrak2. Interstitial glucose measured by FreeStyle Libre 2 continuous interstitial glucose monitor.

A total of 364 paired BG and IG data points were recorded from 9 cats during periods of hypoglycemia and euglycemia (range, 29‐120 mg/dL). During periods of both stable and unstable glycemia (n = 364), BG and IG correlated moderately (r = 0.69, 95% confidence interval [CI], 0.63‐0.74; *P*  < .0001, Figure 1). In the Parkes Error Grid analysis, 100% of IG results fell in zones A and B (333 in zone A [91.5%], 31 in zone B [8.5%]), with none in other zones (Figure 2). Only 73% of all IG results were within 15 mg/dL (when BG ≤100 mg/dL) or 15% (when BG > 100 mg/dL) of their paired BG results. Overall bias between BG and IG was −2.5 ± 14.9 (95% CI, −31.7 to 26.7) mg/dL and positively correlated (r = 0.4) with the magnitude of glycemia (Figure 3). Measures of analytical accuracy are presented in Table 1.

**TABLE 1:** Measures of analytical accuracy in paired blood glucose (BG) and interstitial glucose (IG) concentrations at all rates of change (n = 364) and during stable glycemia (n = 217).

Parameter	All data (n = 364)	Stable data (n = 217)
Mean absolute relative difference (MARD) (%)	18.2	15.4
Median absolute relative difference (mARD) (%)	14.9	13.7
Mean relative difference (MRD) (%)	0.9	0.8
Mean absolute difference (MAD)	11.8	10.3

**FIGURE 4:**
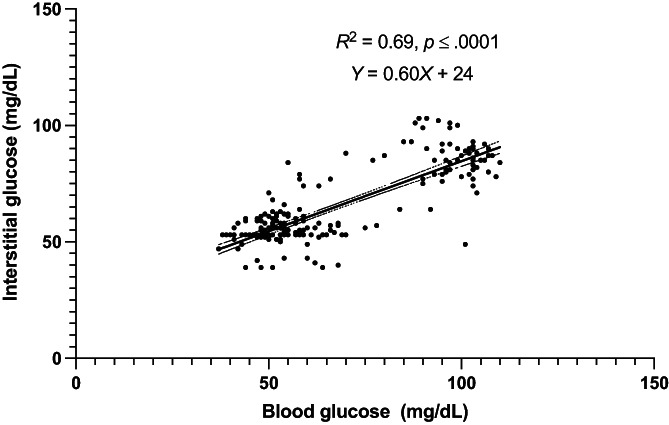
Correlation between blood glucose (BG) and interstitial glucose (IG) concentrations in healthy cats (n = 9) in hypo‐ and euglycemia during stable BG. The middle, solid line represents best fit with dashed lines representing the 95% CI of the best fit. Blood glucose measured by AlphaTrak2. Interstitial glucose measured by FreeStyle Libre 2 continuous interstitial glucose monitor.

**FIGURE 5:**
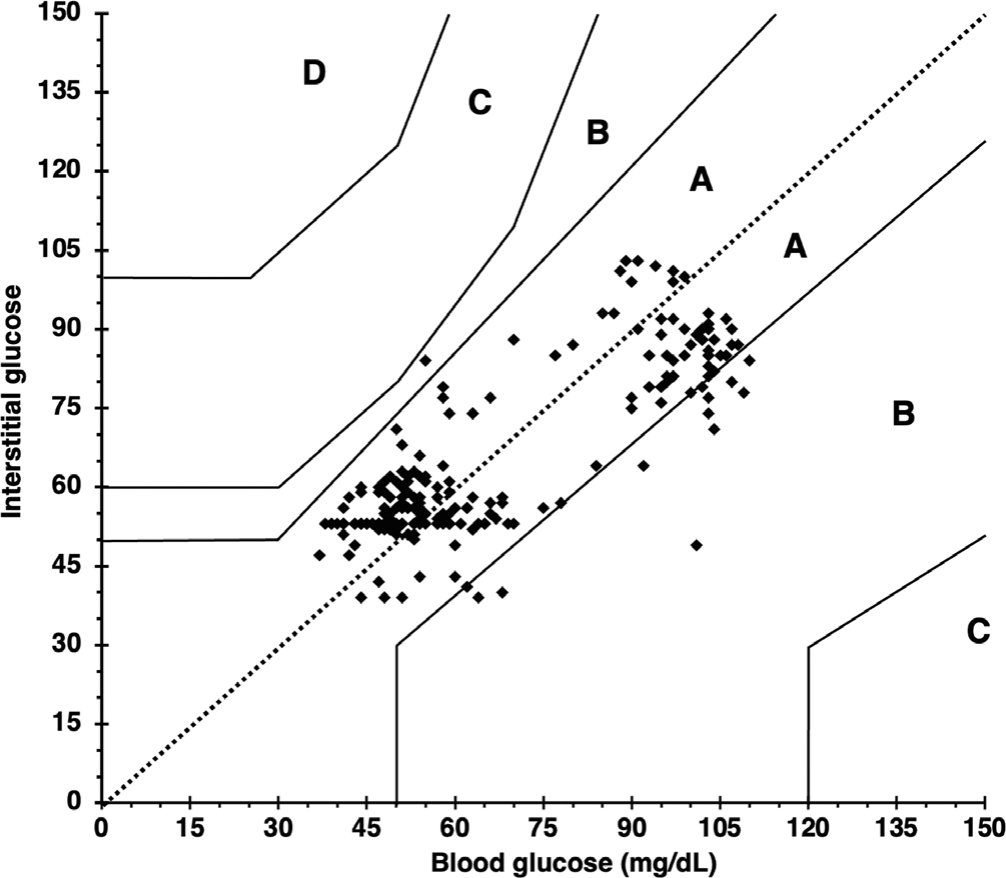
Parkes Error Grid analysis exhibiting excellent clinical accuracy of FSL2 at hypo‐ and euglycemia in healthy cats (n = 9) during steady blood glucose (BG) concentrations. All data points fall within zone A (indicating no change in clinical action) or zone B (indicating change in clinical action unlikely to affect outcome), with 94% (n = 204) data points in zone A and 6% (n = 13) in zone B. BG, measured by AlphaTrak2. IG, interstitial glucose as measured by FSL2, FreeStyle Libre 2 continuous interstitial glucose monitor.

Of the entire data set of 364 pairs, 217 paired values occurred during periods of stable glycemia (≤1 mg/dL/min change in BG over 10 minutes). In this subset, BG and IG correlated strongly (r = 0.83; 95% CI, 0.78‐0.87; *P*  < .0001; Figure 4) and 100% of IG results were again in zones A and B of the Parkes Error Grid. Of these, 204 pairs fell in zone A (94.0%) and 13 in zone B (6.0%; Figure 5). Seventy‐five percent of IG results were within 15 mg/dL (when BG ≤100 mg/dL) or 15% (when BG >100 mg/dL) of their paired BG results during stable glycemia.

**FIGURE 6:**
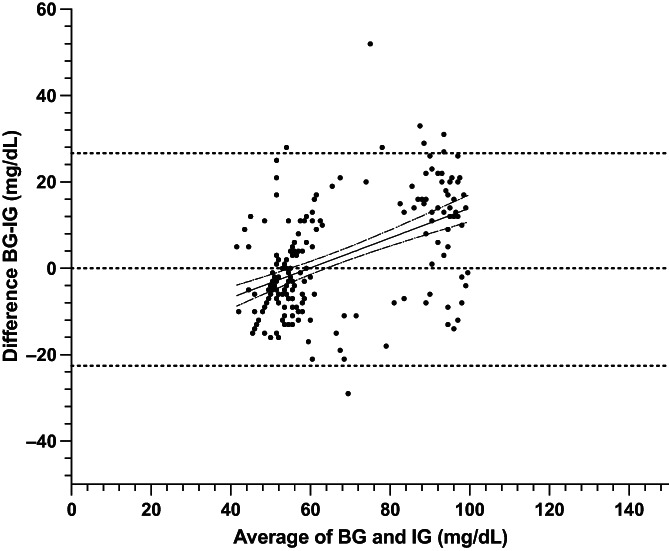
Bland Altman plot of agreement between blood glucose (BG) and interstitial glucose (IG) concentrations in hypo‐ and euglycemia in healthy cats (n = 9) during steady BG. The solid line represents best fit with dashed lines representing the 95% CI of the best fit. BG, blood glucose as measured by AlphaTrak2. IG, interstitial glucose as measured by FreeStyle Libre 2 continuous interstitial glucose monitor.

**FIGURE 7:**
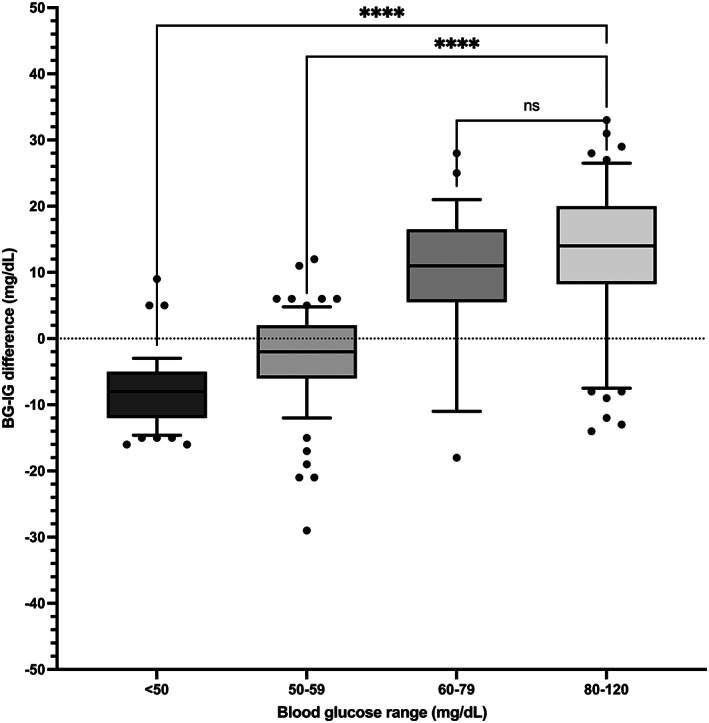
Difference between blood glucose (BG) and interstitial glucose (IG) concentrations, stratified based on BG range in healthy cats (n = 9) during steady BG. Central horizontal lines represent median values, boxes represent quartiles, whiskers represent 10% to 90% percentiles, and asterisks represent statistically significant differences [Correction added after first online publication on 8 August 2023. Figure 7 has been replaced.].

In stable glycemia and across the entire range of hypo‐ and euglycemia, the bias between BG and IG was 2.0 ± 12.6 (95% CI, −22.6 to 26.6) mg/dL and positively correlated (r = 0.5) with the magnitude of glycemia (Figure 6). Interstitial glucose concentration underestimated BG in euglycemia and mild hypoglycemia by a mean of 12.9 ± 12.2 in the 80 to 120 mg/dL range (n = 64) and 8.8 ± 11.2 in the 60 to 79 mg/dL range (n = 29; *P* = .1; Figure 7). Interstitial glucose concentration instead overestimated BG by a mean of 3.2 ± 7.4 in the 50 to 59 mg/dL range (n = 71; *P* < .0001) and 7.8 ± 5.2 in the 29 to 49 mg/dL range (n = 53; *P* < .0001; Figure 7).

Glycemia was noted to fluctuate intermittently in association with auditory and visual stimuli, independent of change in glucose infusion rate. Overt signs of neuroglycopenia were not observed. Adverse events were characterized by 8 episodes of vomiting (from collective data at all rates of glycemic change). Five episodes occurred during severe hypoglycemia (37‐49 mg/dL), 2 during marked hypoglycemia (51‐53 mg/dL), and 1 during euglycemia (106 mg/dL).

## 4 DISCUSSION

The increasing use of continuous IG monitors represents a paradigm shift in veterinary diabetic management. Although CGMs provide an unparalleled ability to monitor glucose trends, their accuracy has not been fully assessed in cats in the hypoglycemic range. Our data suggest a small and proportional bias in IG values in the hypoglycemic range. Interstitial glucose tends to underestimate BG in euglycemia, as previously reported,^7^, ^12^ but this difference is reduced as BG decreases. In marked hypoglycemia, IG tends to overestimate BG in cats. This strays from previously reported fixed IG underestimation of BG, assumed to be present at all glycemic ranges.^7^

Acceptable CGM accuracy traditionally is determined by comparison to BG measurements, in adherence with the International Organization for Standardization (ISO) 15 197:2013 guidelines for humans. These guidelines were established for point‐of‐care BG meters, not for CGMs that measure IG.^8^ According to these guidelines, clinical accuracy is defined as having 99% of IG results fall within zones A and B of the Parkes Error Grid. In these zones, whether glucose measurement is performed by the gold standard or by the method under study, the clinical action is identical (Zone A) or similar enough (Zone B) so that it is unlikely to affect patient outcome.^16^ In contrast to clinical accuracy, ISO guidelines define acceptable analytical accuracy as having 95% of IG measurements fall within 15 mg/dL (when BG <100 mg/dL) or 15% (when BG >100 mg/dL) of the reference BG method. However, because IG and BG measure glucose in different compartments that are affected by different physiological processes, analytical accuracy is not expected for CGMs and generally is not met with any form of IG measurement.^7^, ^12^ Similar to previous veterinary CGM studies utilizing ISO 15197:2013 guidelines, standards for clinical (but not analytical) accuracy were met in our study of FSL2. In contrast to previous studies, we demonstrate this level of clinical accuracy in the hypoglycemic range and during low and high rates of glycemic change.^7^, ^11^, ^12^, ^18^, ^19^

The accuracy of FSL2 during hypo‐ and euglycemia in cats is reported here with 364 paired BG‐IG measurements in total, 242 of them in the hypoglycemic range (BG <70 mg/dL), with 143 of those during periods of stable glycemia. Previously published data in cats have been limited to a maximum of 10 paired readings at BG <70 mg/dL.^5^ Comparable to previous studies using the FSL in cats, a strong correlation between BG and IG during stable glycemia was identified. However, for the entire data set that includes both stable and less stable BG, we report a more moderate correlation compared to previous studies. Direct comparison between studies is limited by difference in hypoglycemic sample size (maximum n = 10),^5^ method of reference glucose measurement (point‐of‐care glucometer vs hexokinase‐based laboratory measurement), population (client‐owned diabetic vs purpose‐bred cats), sampling environment and frequency (at‐home, in‐hospital, or research laboratory), and FSL model (FSL1 in previous studies).^1^, ^5^, ^7^, ^12^ Rate of glycemic change in previous studies was largely unknown because data were obtained from client‐owned diabetics during routine and relatively infrequent BG monitoring. In 1 study of purpose‐bred cats in which data were obtained with frequent enough BG monitoring, the subset of data that represented a high rate of BG change was excluded from analysis of IG vs BG correlation and bias.^7^ It is thus impossible to assess whether the lower correlation we report here is because of the range in which it was measured (hypo‐ and euglycemia vs hyperglycemia), the range of rate of change BG, or the method (FSL2 vs FSL1).

The induced hypoglycemia inherent to our study provided a unique opportunity to safely and humanely collect an unprecedented volume of data in this glycemic range. In the clinical setting, some of the difference between BG and IG is caused by the time required for glucose concentrations to stabilize and reach equilibrium between the interstitium and the blood. Maintaining a stable BG by using the glucose clamp method allowed for more accurate assessment of bias in IG measurement in reference to BG by minimizing the confounding effect of lag time required for equilibrium between compartments.

Previously reported lag times vary widely based on method used to induce change in BG, whether BG is increasing or decreasing, and the CGM device used.^6^ Rapid, high‐dose (0.5 g/kg) IV glucose infusion in cats results in a lag of 5 to 10 minutes from injection to the first upward change in IG, and 30 to 45 minutes to the maximum IG recorded.^7^, ^12^ Lag time is prolonged in patients with decreased interstitial tissue perfusion, such as older or dehydrated animals,^7^, ^10^ and when changes in BG are rapid and of large magnitude. The cats used in our study were clinically euhydrated and of young‐adult age. The magnitude of BG change was limited to the narrow hypoglycemic range tolerated by live animals. Sympathetic responses inherent to conscious cats causing unpredictably rapid BG change were minimized by restraint‐free handling and acclimatization to study personnel and laboratory conditions.

The inclusive data set (paired readings at both stable and unstable glycemia) might be more representative of the clinical setting than the stable‐only subset. Rate of glycemic change in insulin‐dependent patients depends upon variable and often unpredictable absorption of exogenous insulin from the SC depot. In the clinical setting, users of FSL2 are prompted by trend arrows to confirm IG readings with BG measurements when the rate of IG change is high. We defined stable glycemia (≤1 mg/dL/min) more strictly than the FSL manufacturer's definition (≤2 mg/dL/min) for the purpose of accurately assessing the bias between IG and BG. Considering our entire data set, IG readings obtained during high rates of glycemic change or in dehydrated and older cats may require additional BG validation.

Our results contradict the common assumption that CGMs such as the FSL2 report IG with a fixed bias, underestimating IG readings at all glycemic levels. A proportional bias instead was found, with IG overestimation of BG in severe hypoglycemia. This glycemic range‐dependent bias likely explains the discrepancy between our small overall bias of −2.5 mg/dL and the previously reported larger bias of −23 mg/dL. This prior data was derived from paired CGM and BG results in the 50 to 672 mg/dL glycemic range, but direct comparison is limited by few paired data points during hypoglycemia and by use of the FSL1.^7^, ^12^ Importantly, in people the FSL2 is considered substantially more accurate than the FSL1 in the hypoglycemic range.^14^

The small overestimation of BG by FSL2 reported here occurred in a critical glycemic range where clinical action may result in immediate and even life‐threatening consequences. As must be explained to owners, however, isolated low IG (or BG) readings are rarely of concern unless clinical signs of hypoglycemia are noted. Subclinical hypoglycemic nadirs might instead warrant future insulin dosing adjustments, potentially after BG confirmation depending on the clinical context.

Vomiting was correlated with hypoglycemia in our study using fasted, healthy cats. Because overt central neurologic signs were not observed, the presence of vomiting as a more sensitive marker of clinical hypoglycemia in cats warrants further study. It may be prudent to warn caretakers to monitor for vomiting as an indication to check BG and feed a small, carbohydrate‐rich meal.

Limitations of our study include exclusive use of nondiabetic cats with experimentally‐induced hypoglycemia for data collection. Hypoglycemia was induced by infusion of regular insulin, resulting in rapid BG shifts that might or might not be comparable to what is seen with intermediate‐ and long‐acting insulin formulations used in the home environment. Although the physiologic basis of BG and IG discrepancy is expected to be the same regardless of cause of hypoglycemia, further study is required to validate these findings in client‐owned diabetic cats, in insulin‐independent hypoglycemic syndromes, and in other species.

In conclusion, clinical action taken in response to BG measurements in the hypoglycemic range carry the potential for critical consequence. Thus, use of a BG proxy such as IG requires understanding of its limitations. Comprehension of the glycemia‐dependent bias in FSL2 IG enables clinicians to optimize the safety of its use, mitigating the inherent discrepancy of glucose measurements between body fluid compartments. Clinicians should be aware of the proportional, glycemic‐dependent bias between IG and BG measurements. Clinicians should be cautioned that although the FSL2 IG monitor tends to underestimate BG in most of the euglycemic and hyperglycemic range in cats, it may overestimate BG in hypoglycemic ranges <60 mg/dL. Interstitial glucose readings in the severely hypoglycemic range should be approached with caution and interpreted as potentially representing BG at an equal or lower concentration.

## CONFLICT OF INTEREST DECLARATION

Authors declare no conflict of interest.

## OFF‐LABEL ANTIMICROBIAL DECLARATION

Authors declare no off‐label use of antimicrobials.

## INSTITUTIONAL ANIMAL CARE AND USE COMMITTEE (IACUC) OR OTHER APPROVAL DECLARATION

Approved by the IACUC of the University of Florida (protocol #202011101).

## HUMAN ETHICS APPROVAL DECLARATION

Authors declare human ethics approval was not needed for this study.
